# Draft Genome Sequence of Shewanella algidipiscicola H1, a Highly Chromate-Resistant Strain Isolated from Mediterranean Marine Sediments

**DOI:** 10.1128/MRA.00905-18

**Published:** 2018-08-30

**Authors:** Hiba Baaziz, Olivier N. Lemaire, Cécile Jourlin-Castelli, Chantal Iobbi-Nivol, Vincent Méjean, Radia Alatou, Michel Fons

**Affiliations:** a Aix Marseille Université, CNRS, BIP UMR 7281, Marseille, France; b Université des Frères Mentouri Constantine 1, Laboratoire de Biologie Moléculaire et Cellulaire, Constantine, Algeria; University of Maryland School of Medicine

## Abstract

The ability of different Shewanella spp. to convert heavy metals and toxic substances into less toxic products by using them as electron acceptors has led to their use in environmental clean-up strategies. We present here the draft genome sequence of Shewanella algidipiscicola H1, a strain resistant to high concentrations of chromates.

## Abstract (precis)

The ability of different Shewanella spp. to convert heavy metals and toxic substances into less toxic products by using them as electron acceptors has led to their use in environmental clean-up strategies.

## ANNOUNCEMENT

Most Shewanella spp. are isolated from marine environments. Their survival in various habitats and consequently their wide distribution in nature partly rely on their capability to utilize an extended array of final electron acceptors, including metals (1–3). In the environment, chromium can be present as chromate [Cr(VI)], a powerful oxidant found in soluble oxyanion forms. Cr(VI) is highly toxic because it rapidly enters into the cytoplasm, where it generates reactive oxygen species (ROS) that are deleterious for cells (4). Bacteria can repair chromate-induced damage. They have also developed different resistance mechanisms that directly target Cr(VI), including downregulation of chromate ion uptake, their efflux from the cell cytoplasm, and reduction of Cr(VI) into Cr(III) (5, 6). Under aerobic conditions, Cr(VI) reduction is usually achieved in the cytoplasm by chromate reductases of type I (ChrR from Pseudomonas putida or YieF from Escherichia coli) or type II (NfsA from E. coli) (7, 8).

The S. algidipiscicola strain H1 was isolated from the muddy sediment of Stora Harbor (36°54′06.9″N 6°52′45.4″E, at Skikda on the Mediterranean Algerian coast) sampled from the uppermost 3 cm. The strain H1 was grown aerobically in lysogeny broth (LB) medium supplemented with NaCl (15 g/liter final concentration) at 28°C under stirring conditions. Total DNA was extracted using the GenElute bacterial genomics kit (Sigma-Aldrich). Genome sequencing of S. algidipiscicola H1 was carried out at the Molecular Research LP (MR DNA) Laboratory (USA). The library was prepared using the Nextera DNA Sample Preparation kit (Illumina) following the manufacturer’s user guide. The sample was diluted accordingly to achieve the recommended DNA input of 50 ng at a concentration of 2.5 ng/µl. Subsequently, the sample underwent the fragmentation and addition of adapter sequences used for 5 PCR cycles. The average library size was 708 bp. The library was then pooled in equimolar ratios, and 2 nM, and 10 pM of the library pool were clustered with the cBot system (Illumina) and paired-end sequenced for 300 cycles with the HiSeq 2500 system (Illumina).

The genome assembly was generated at the MR DNA laboratory with the SeqMan NGen software assembler (DNASTAR). A total of 355,087,028 reads were assembled into 22 contigs, with a total length of 4,140,480 bp. Using the MaGe (Magnifying Genomes) Web-based interface (see http://www.genoscope.cns.fr/agc/microscope/mage/), a preliminary analysis indicates that the draft genome sequence contains 3,857 predicted genes. Among them, 3,678 are potential protein-coding genes (CDSs) without artifacts, 13 are rRNAs, and 90 are tRNAs. Until now, only one Shewanella sp. genome from the Mediterranean environment was available, that of S. woodyi ATCC 51908.

Preliminary results show that strain H1 is able to grow in the presence of 3 mM chromate under previously described semiaerobic conditions, whereas the model strain, S. oneidensis MR1, stops growing when the Cr(VI) concentration is more than 0.5 mM (9).

The draft genome sequence of S. algidipiscicola H1 could thus provide novel information about chromate resistance and reduction systems.

### Data availability.

The results obtained from this whole-genome shotgun project have been deposited at the European Nucleotide Archive database under accession number OXJV01000000 (assembly contigs OXJV01000001 through OXJV01000022).
